# Loose PEG Tube Leading to Peristomal Leakage and Peritonitis, a Case Report

**DOI:** 10.21980/J8HS7T

**Published:** 2020-04-15

**Authors:** Connie Au, Toby Myatt

**Affiliations:** * University of California, Irvine, Department of Emergency Medicine, Orange, CA

## Abstract

We present the case of a 37-year-old male with history of amyotrophic lateral sclerosis (ALS) and recent percutaneous endoscopic gastrostomy (PEG) tube placement presented with abdominal pain, nausea, vomiting, abdominal distention, tenderness and guarding. Given the concern for peritonitis, upright/decubitus chest X-ray was obtained and showed bilateral free peritoneal air suggesting possible perforation or peristomal leakage after PEG tube placement. While PEG complication rates are relatively low, clinicians should consider them and remember that chest X-ray can be a very efficient and accurate method of evaluation for possible gastrointestinal perforation, especially in acute emergencies.

**Topics:**

Abdominal/gastrointestinal, peritonitis, perforation, surgical complication.

[Fig f1-jetem-5-2-v7]

**Figure f1-jetem-5-2-v7:**
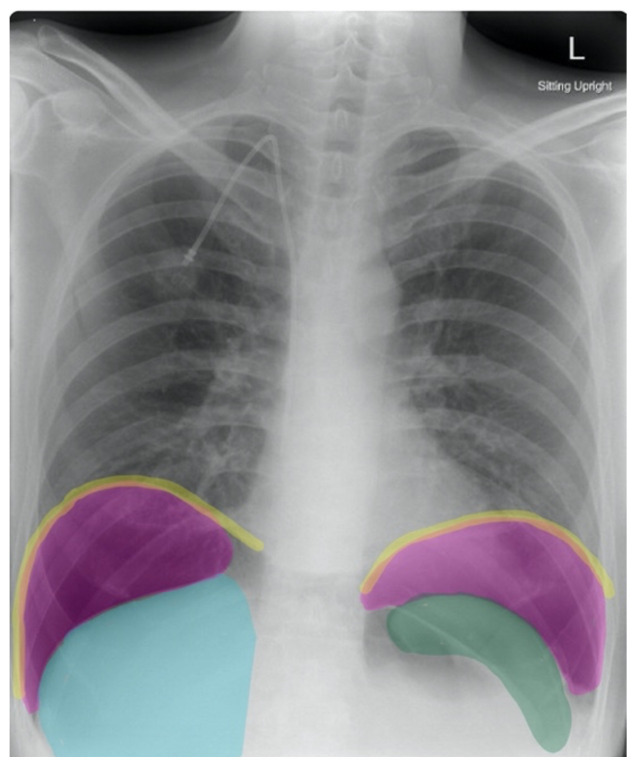

[Fig f2-jetem-5-2-v7]

**Figure f2-jetem-5-2-v7:**
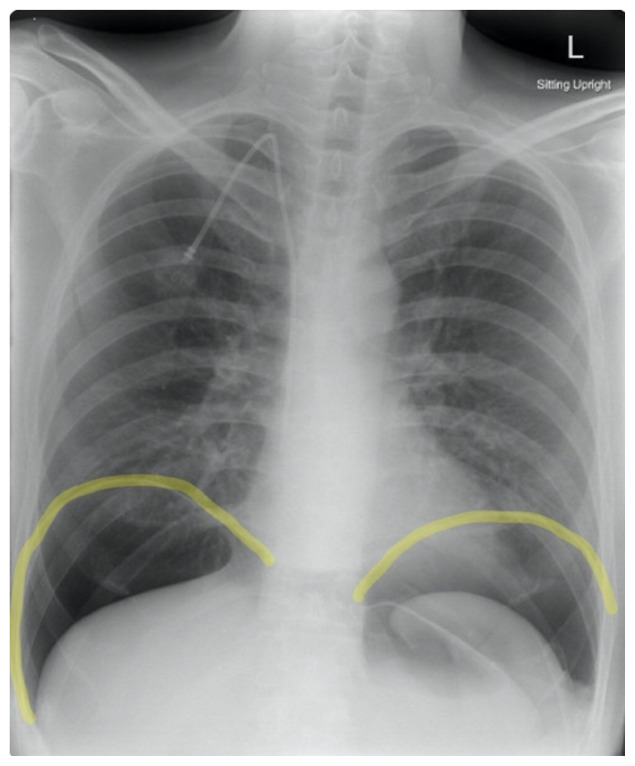

[Fig f3-jetem-5-2-v7]

**Figure f3-jetem-5-2-v7:**
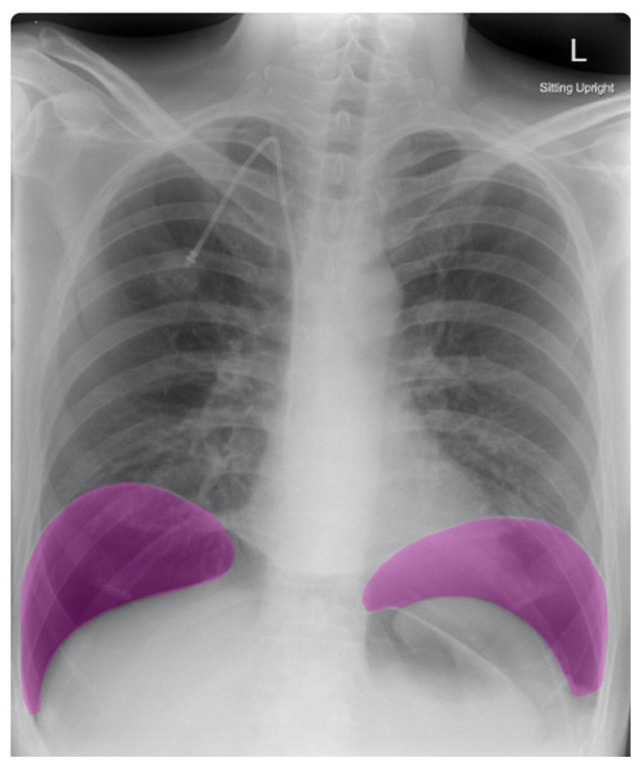

[Fig f4-jetem-5-2-v7]

**Figure f4-jetem-5-2-v7:**
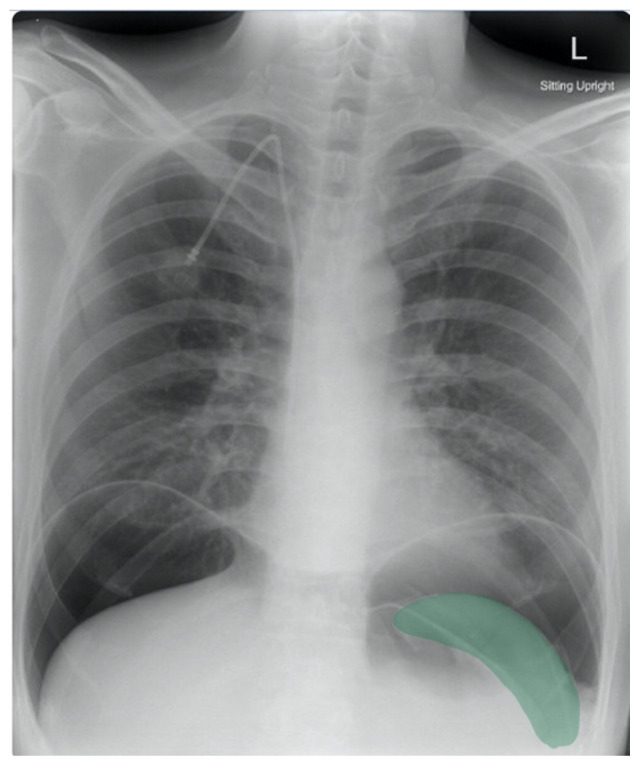

[Fig f5-jetem-5-2-v7]

**Figure f5-jetem-5-2-v7:**
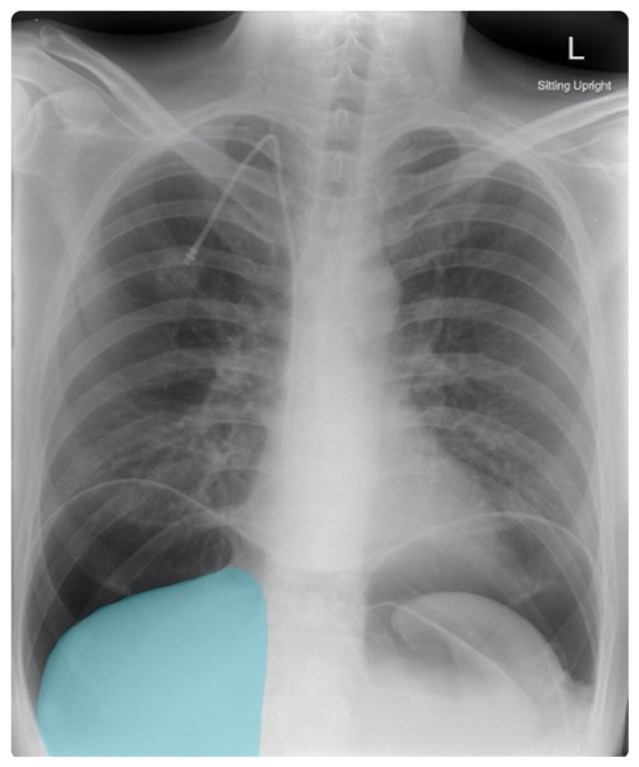

## Introduction

Percutaneous endoscopic gastrostomy (PEG) is a common endoscopic procedure with an overall success rate of 95% to 98% that is performed to provide enteral nutrition, medications, and fluids to patients with oral or esophageal complications.^1^,^2^ The PEG complication rate varies from 16% to 39%.^1^,^3^ Major complications include necrotizing fasciitis, peristomal leakage, buried bumper syndrome, aspiration pneumonia, and internal organ injury.^1^ Minor complications are three times more common than major complications and typically include local wound infection, tube dislodgement, gastric outlet obstruction, and pneumoperitoneum.^1^,^2^

In this case report, we present a patient who came into the emergency department (ED) with severe abdominal pain and diffuse abdominal tenderness after a PEG procedure done earlier that same day at a different hospital facility.

## Presenting concerns and clinical findings

A 37-year-old male with history of ALS and recent PEG tube placement presented with diffuse abdominal pain, nausea, and vomiting for seven hours after PEG placement. Patient had some initial pain after the procedure that was controlled with oral narcotics prior to discharge. After returning home, the patient started experiencing worsening abdominal pain that was no longer controllable with his pain medication. On presentation, his vitals were significant for hypertension and tachycardia. On physical examination, the patient had abdominal distention, diffuse abdominal tenderness with palpation, hypoactive bowel sounds, and guarding, concerning for peritonitis. His laboratory workup revealed an elevated white blood count of 22,600 cells/mm^3^ and an elevated serum total bilirubin of 1.7 mg/dL. Two-view chest X-ray and abdominal computed tomography (CT) were ordered.

## Significant findings

Frontal chest X-ray showed a large radiolucent area (pink highlighted area) underneath the diaphragm (yellow line) and on top of the liver (blue highlighted area) and spleen (green highlighted area) suggestive of pneumoperitoneum possibly caused by gastrointestinal perforation. This large radiolucent area can also be seen underneath the diaphragm in the lateral view chest X-ray. Computed tomography (CT) was not performed due to his physical exam findings and the significant positive findings on chest X-ray. Surgery was consulted and patient was taken emergently to the operating room.

## Patient course

In this case, the patient had a 2-view chest X-ray performed in the emergency department which showed signs of intrabdominal free air. Given the severity and urgency of the patient’s condition, CT was ordered but not completed. Chest X-ray findings, clinical signs, and lab values suggested peritonitis. The patient was rushed to the operating room and had an emergent exploratory laparoscopy. The surgery team found that the PEG tube had come loose and slid out of the stomach into the peritoneum, resulting in leakage of gastric contents and free air. One-hundred and fifty ml of gastric contents were suctioned from the pelvis and above the liver. The PEG tube skin bumper was tightened, and the patient was admitted for observation. The patient did well after surgery and had no further complications. He was given piperacillin/tazobactam during his stay and did not require prolonged hospitalization.

## Discussion

According to previous studies, the incidence of peristomal leakage after PEG placement is 1% – 2%.^4^ Patients with impaired wound healing, such as those with diabetes, malnutrition, and immunodeficiency, are at higher risk for peristomal leakage.^5^ Peritonitis that develops as a result of peristomal leakage is an even more rare occurrence. Development of peritoneal irritation and rebound tenderness differentiate peritonitis from PEG-site infection.^2^ Treatment for peritonitis requires radiographic contrast studies to rule out the presence of a leak.^2^ If no leak is demonstrated, broad spectrum intravenous antibiotics are recommended.^2^ If active leakage is identified after contrast studies in a patient with clinical signs of peritonitis, surgical exploration and broad-spectrum antibiotics are indicated.^2^,^6^,^7^

Although CT is a more sensitive and specific examination for evaluating free air and bowel perforation than chest X-ray, chest X-ray can be obtained more quickly. If there is clinical suspicion of peritonitis or bowel perforation, an upright CXR should be obtained as an initial imaging study. Upright chest X-ray has an increased sensitivity of 85.1% for free air detection as compared to supine chest X-ray, which has a sensitivity of 78.7%.^8^ Similarly, upright chest radiographs have been shown to be able to detect very small amounts of extraluminal free air, with a sensitivity of 70% and a specificity of 93% after 2–3 days post-op.^9^ The presence of free air in chest radiographs has been associated with an increased risk of gastrointestinal perforation, with a positive predictive value of 33%.^9^ These findings suggest that chest X-ray can be a sensitive method of evaluation for free air detection and possible gastrointestinal perforation, especially in an acute emergency.

It should be noted that negative chest X-ray findings do not exclude free air or underlying gastrointestinal pathology. Further imaging studies may be indicated if there is clinical concern for peritonitis. Computed tomography has a sensitivity of 97.2% in detecting the formation and source of peritonitis and is the imaging method of choice for PEG malposition and assessment of peritonitis.^10^,^11^

## Supplementary Information














