# Supplementary figures and images for: Loose PEG Tube Leading to Peristomal Leakage and Peritonitis, a Case Report
Source: J Educ Teach Emerg Med. 2020 Apr 15;5(2):V7–V10. doi: 10.21980/J8HS7T (PMC10332566; doi:10.21980/J8HS7T)

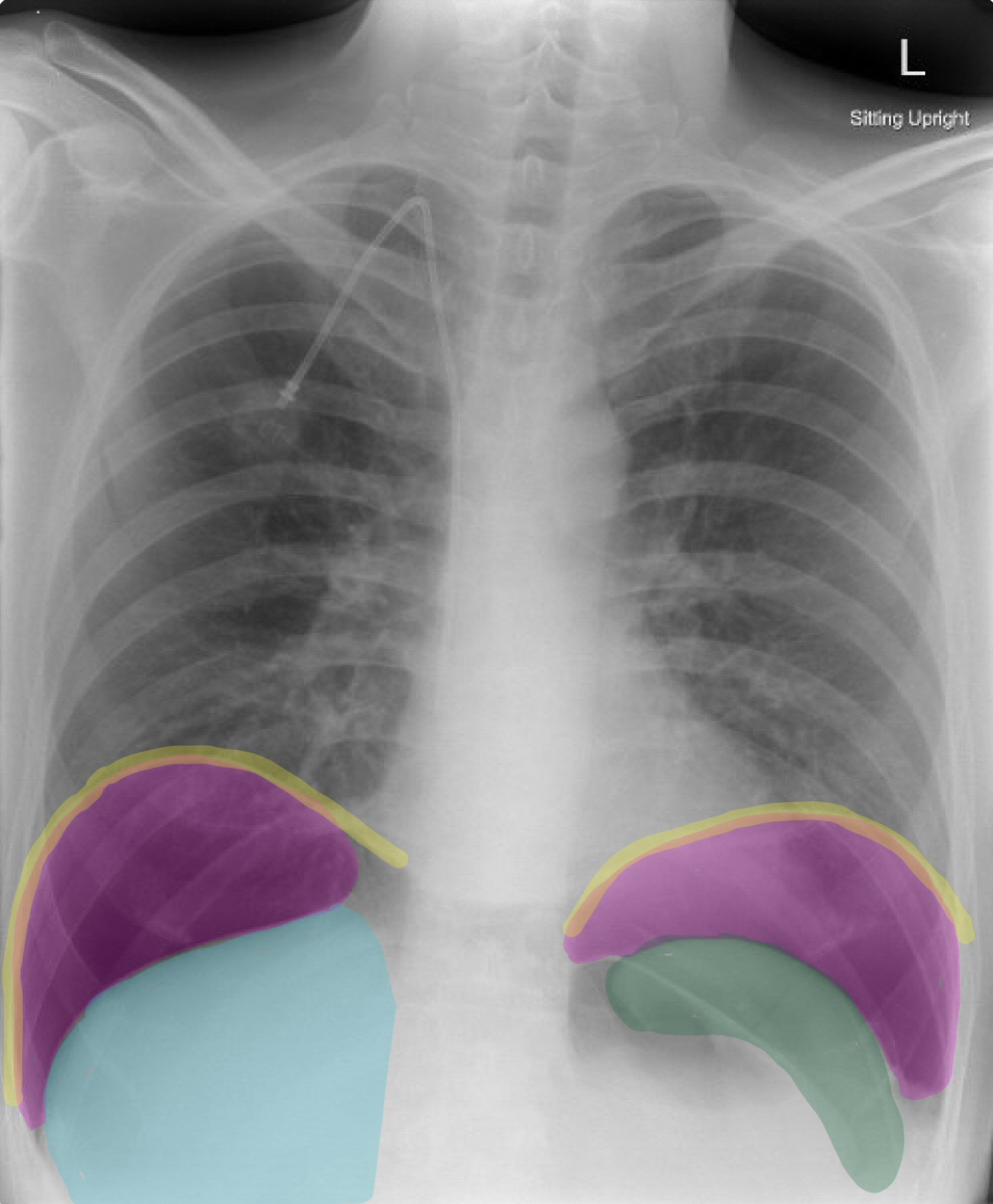

Supplement: Supplementary file 1 [file jetem-5-2-v7-supp1.jpg]

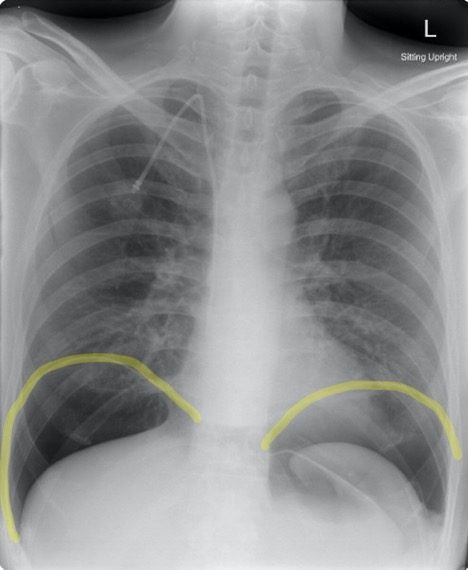

Supplement: Supplementary file 2 [file jetem-5-2-v7-supp2.jpg]

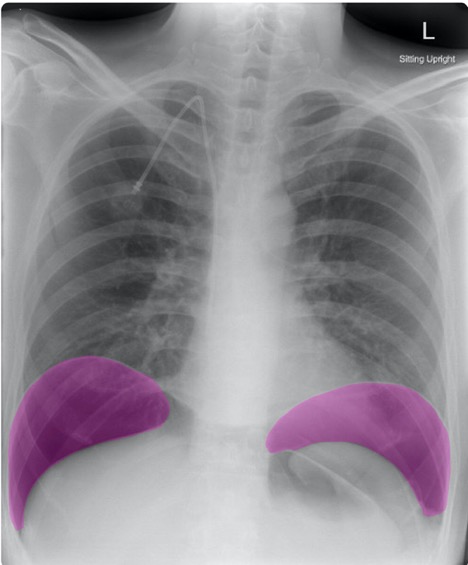

Supplement: Supplementary file 3 [file jetem-5-2-v7-supp3.jpg]

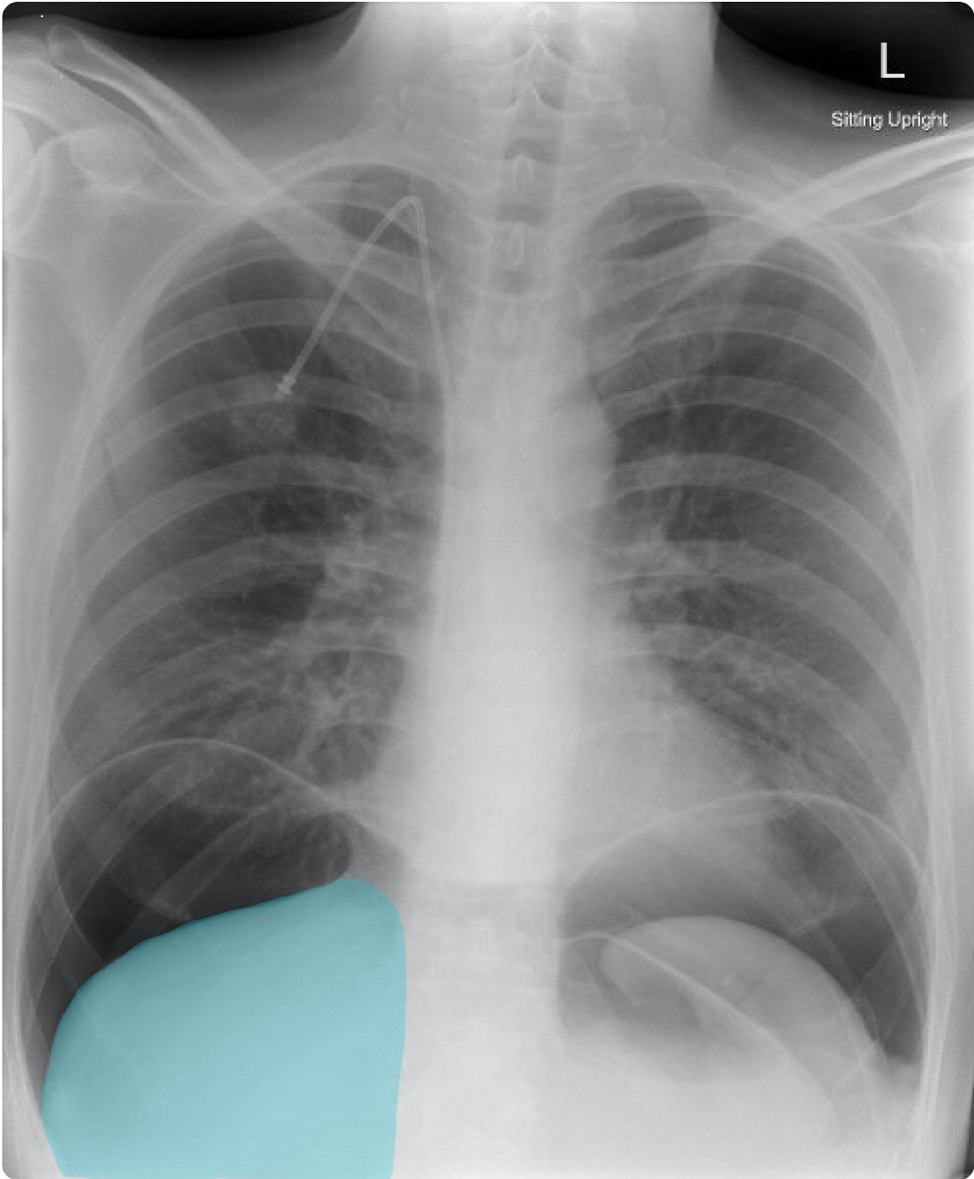

Supplement: Supplementary file 4 [file jetem-5-2-v7-supp4.jpeg]

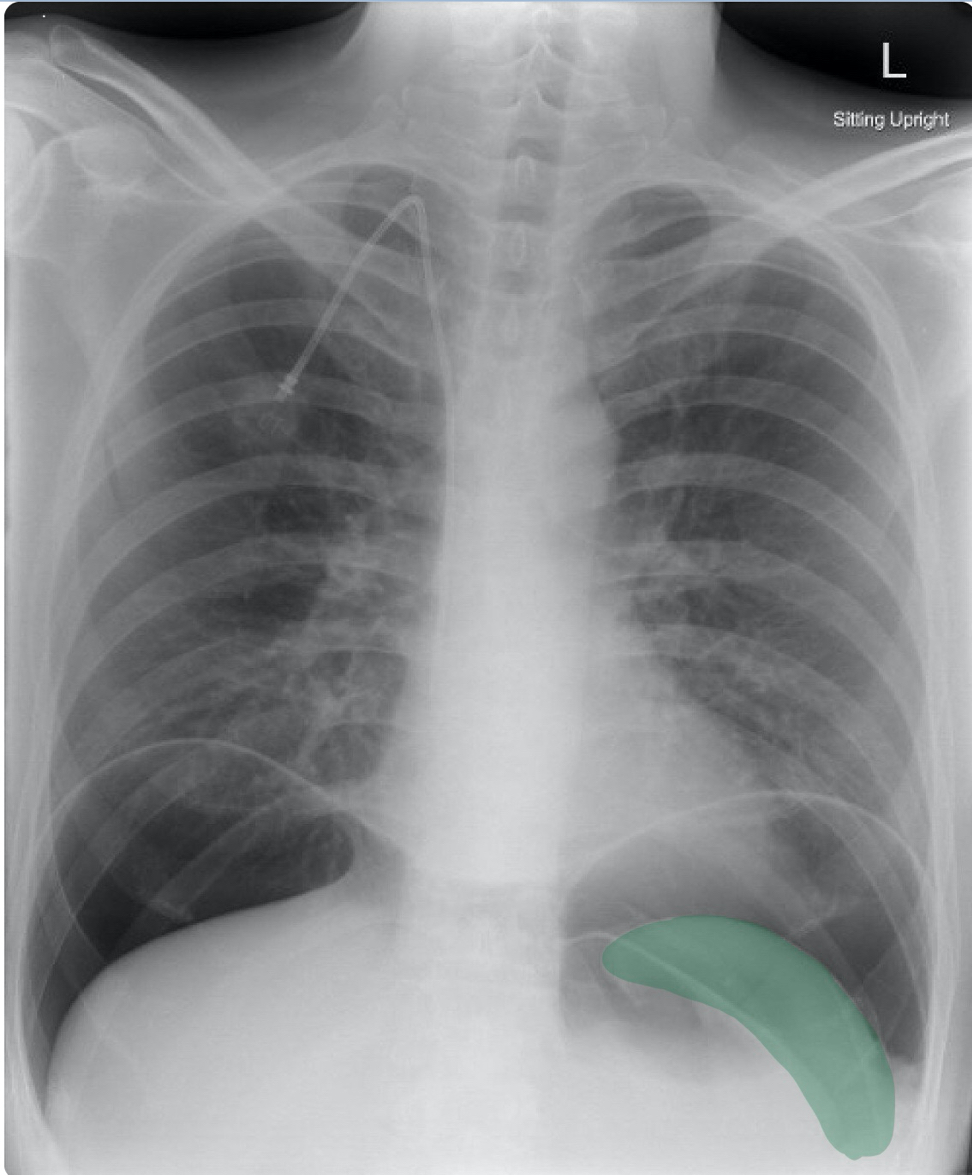

Supplement: Supplementary file 5 [file jetem-5-2-v7-supp5.jpeg]

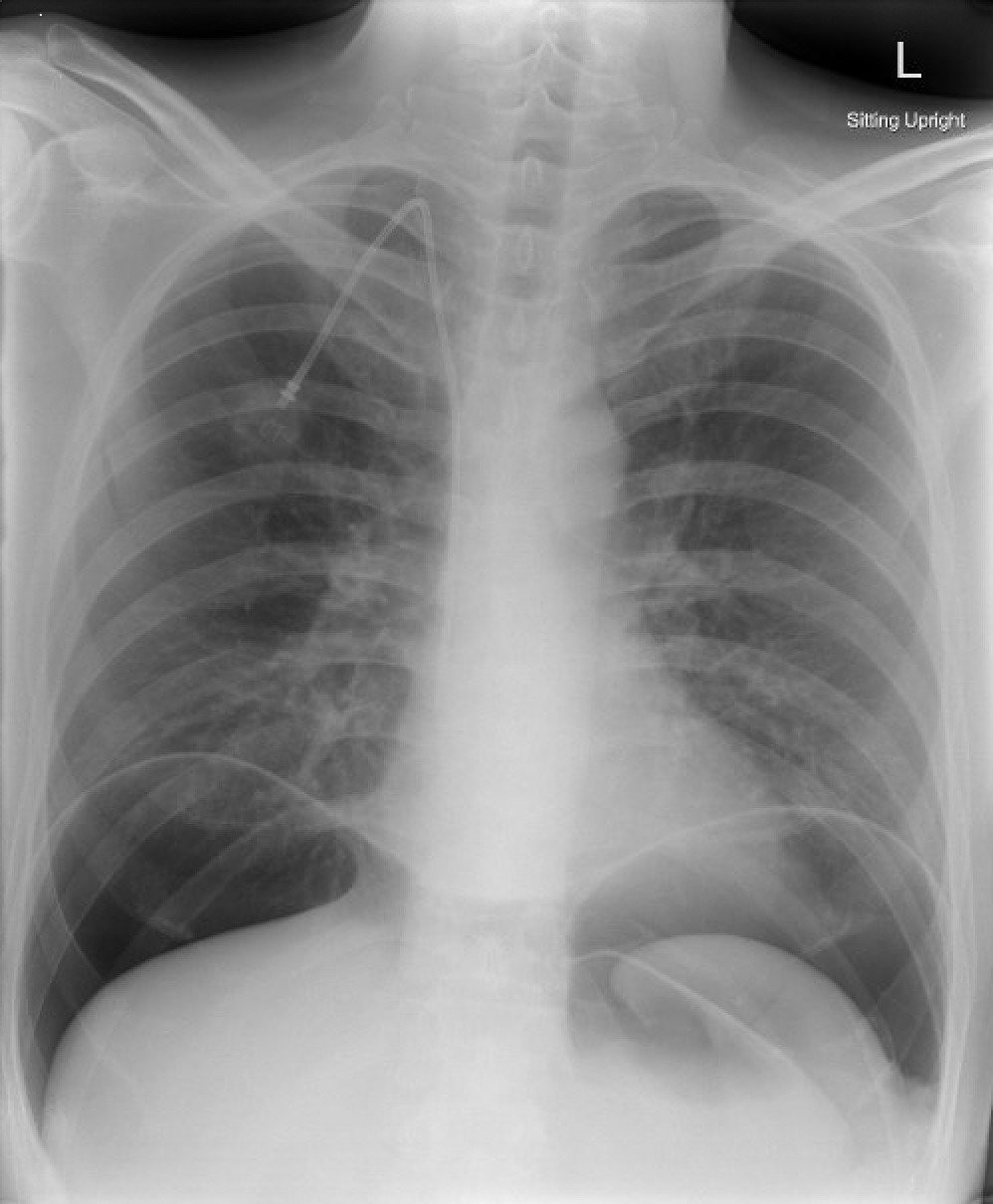

Supplement: Supplementary file 6 [file jetem-5-2-v7-supp6.jpg]

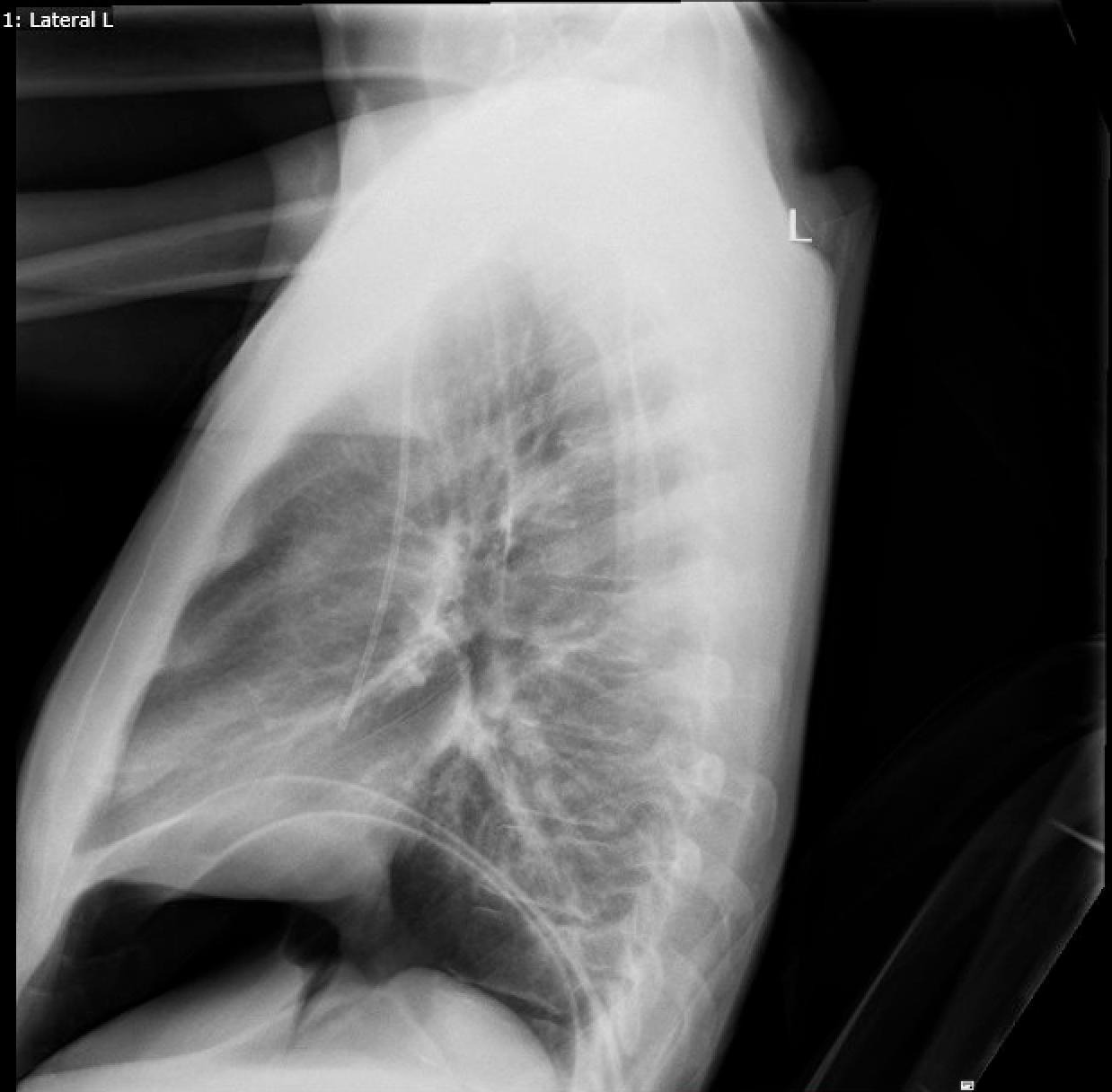

Supplement: Supplementary file 7 [file jetem-5-2-v7-supp7.jpg]
